# Posterior quadratus lumborum block for primary total hip arthroplasty analgesia: a comparative study

**DOI:** 10.31744/einstein_journal/2019AO4905

**Published:** 2019-09-04

**Authors:** Promil Kukreja, Lisa MacBeth, William Potter, Katherine Buddemeyer, Henry DeBell, Hesham Elsharkawy, Hari Kalagara, Andre Wajnsztejn, Eduardo Araujo Pires, Alexandre Leme Godoy-Santos, Ashish Shah

**Affiliations:** 1 Department of Anesthesiology and Perioperative Medicine, University of Alabama, Birmingham, AL, USA.; 2 Department of Orthopaedic Surgery, University of Alabama, Birmingham, AL, USA.; 3 Cleveland Clinic, Anesthesiology Institute, Cleveland, OH, USA.; 4 Hospital Israelita Albert Einstein, São Paulo, SP, Brazil.; 5 Department of Orthopedics and Trauma, Hospital das Clinicas, Faculdade de Medicina, Universidade de São Paulo, São Paulo, SP, Brazil.

**Keywords:** Analgesia, Anesthetics, local, Arthroplasty, replacement, hip, Pain, postoperative, Analgesics, opioid

## Abstract

**Objective:**

To compare analgesia and opioid consumption for patients undergoing primary total hip arthroplasty with preoperative posterior quadratus lumborum block with patients who did not receive quadratus lumborum block.

**Methods:**

The medical records of patients undergoing unilateral total hip arthroplasty between January 1st, 2017 and March 31, 2018 were reviewed, and 238 patients were included in the study. The primary outcome was postoperative opioid consumption in the first 24 postoperative hours. Secondary outcomes were intraoperative, post anesthesia care unit, and 48-hour opioid consumption, postoperative pain Visual Analog Scale scores, and post-anesthesia care unit length of stay. Primary and secondary endpoint data were compared between patients undergoing primary total hip arthroplasty with preoperative posterior quadratus lumborum block with patients who did not receive quadratus lumborum block.

**Results:**

For the patients who received quadratus lumborum block, the 24-hour total oral morphine equivalent (milligram) requirements were lower (53.82mg±37.41), compared to the patients who did not receive quadratus lumborum block (77.59mL±58.42), with p=0.0011. Opioid requirements were consistently lower for the patients who received quadratus lumborum block at each additional assessment time point up to 48 hours. Pain Visual Analog Scale scores were lower up to 12 hours after surgery for the patients who received a posterior quadratus lumborum block, and the post-anesthesia care unit length of stay was shorter for the patients who received quadratus lumborum block.

**Conclusion:**

Preoperative posterior quadratus lumborum block for primary total hip arthroplasty is associated with decreased opioid requirements up to 48 hours, decreased Visual Analog Scale pain scores up to 12 hours, and shorter post-anesthesia care unit length of stay. Level of evidence: III

## INTRODUCTION

Total hip arthroplasty (THA) is one of the most common orthopedic procedures in the United States, with approximately 300 thousand surgeries performed annually, and the numbers are expected to rise with an increase in the ageing population.^1^ In the era of fast-track protocols for total joint arthroplasties, there seems to be no gold standard regimen for post-operative pain management.^1^

The quadratus lumborum block (QLB) was initially described in 2007, as a posterior transversus abdominis plane (TAP) block to provide satisfactory analgesia after abdominoplasties.^2^ There are many approaches to QLB, with the local anesthetic deposited laterally, posteriorly or anteriorly (transmuscle) in relation to the quadratus lumborum muscle.^3^

Since the initial description of the QLB, it has been used for postoperative analgesia for abdominal surgeries, including caesarean section, inguinal hernia repair, and laparotomy.^4 - 6^ Additionally, there are case reports of satisfactory postoperative analgesia after THA.^7 - 11^ For patients undergoing hemiarthroplasty for femoral neck fracture, lateral QLB has resulted in lower Visual Analog Scale (VAS) pain scores, and less opioid use than in femoral nerve blocks.^12^ The underlying possible mechanisms of action for QLB leading to hip analgesia may be direct spread of local anesthetics to the nerve roots and branches of the lumbar plexus.^13 - 15^

## OBJECTIVE

To evaluate the clinical analgesic effects on patients undergoing posterior quadratus lumborum block in the preoperative period of primary total hip arthroplasty considering opioid consumption and Pain Visual Analog Scale scores.

## METHODS

This retrospective study was approved by the Institutional Review Board (300000976) in accordance with the Declaration of Helsinki. We searched the hospital billing records of our institution, University of Alabama at Birmingham, for all total hip arthroplasties that occurred between January 1st, 2017 and March 31, 2018, using International Statistical Classification of Diseases and Related Health Problems Procedure Coding System (ICD-10 PCS) codes. The search identified 559 arthroplasties and we used random sampling to select patients. The cases undergoing THA revision, anterior approach for THA and post-operative QLB were excluded, and only 355 patients who underwent primary THA, with or without posterior QLB prior to surgery remained. In addition, 117 patients with incomplete data were excluded from the study, thus, leaving 238 medical records to be analyzed.

The study compared patients who received posterior QLB prior to primary THA to patients who underwent primary THA without a block. Data on the anesthesia type used, the presence or absence of a posterior QLB, post-anesthesia care unit (PACU) admission time, and PACU discharge time were also collected. Inpatient post-operative pain scores (zero to 10 VAS) and inpatient opioid use were collected for up to 48 hours after surgery at the following time points: admission at PACU, discharge from PACU, 24 hours, and 48 hours after surgery starting time. Total opioid use was converted into oral morphine equivalent (mg) units. Pain VAS scores closest to the time points of interest were collected. The primary study outcome was 24-hour post-operative opioid consumption. Secondary outcomes included opioid consumption intraoperatively, at the PACU and 48 hours postoperatively; VAS pain scores at PACU admission and discharge, 12, and 48 hours postoperatively; and PACU length of stay.

**Figure 1:**
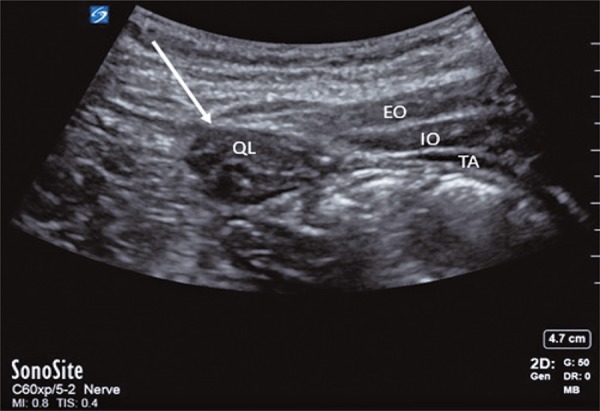
Ultrasound guided approach to posterior quadratus lumborum block. White arrow indicating posterior border of quadratus lumborum muscle

EO: external oblique; IO: internal oblique; TA: transversus abdominis; QL: quadratus lumborum.

Posterior QLB were performed in the preoperative block area in the lateral decubitus position, with standard monitoring of pulse oximetry, non-invasive blood pressure monitoring and eletrocardiogram. Using a curvilinear low frequency ultrasound probe ( Figure 1 ), the posterior QLB was performed using an in-plane lateral to medial approach with 20mL 0.25% bupivacaine with 1:400 concentration epinephrine. These patients then underwent THA under general or spinal anesthesia.

All demographic and clinical variables with continuous measures were expressed as means and standard deviations; categorical variables were expressed as proportions. For non-normal data, the NPAR1WAY SAS software was used for nonparametric tests to provide a standard analysis of variance. The unequal sample sizes were adjusted to maximize the statistical power. The distribution of the continuous variables was examined using the Kolmogorov-Smirnov test. For normally distributed data, the one-way Analysis of Variance (ANOVA) and Student’s *t* test were used to compare groups of data. For not normally distributed data, the Kruskal-Wallis test and Wilcoxon test were used for comparisons. χ^2^ and Fisher’s exact test were used to analyze categorical variables. For all comparisons, p-values of ≤0.05 (two-sided) were considered statistically significant. Student’s *t* test and the ANOVA were used to compare opioid consumption, VAS scores and PACU length of stay.

## RESULTS

**Figure 2:**
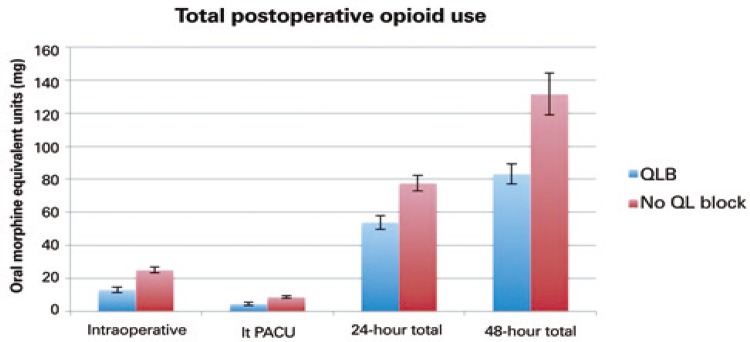
Total opioid consumption in oral morphine equivalent units (mg) at different time points for patients undergoing total hip arthroplasty (with standard error bars)

PACU: post anesthesia care unit; QLB: quadratus lumborum block.

A total of 238 patients were assessed in the study, 79 received a posterior QLB and 159 did not receive a block. For patients receiving a posterior QLB, the 24-hour total oral morphine equivalents (mg) required were 53.82mg±37.41, compared to the Control Group, 77.59mg±58.42 (mean±stadard deviation − SD), with p=0.0011. Opioid requirements were consistently significantly lower for QLB recipients at each time point studied: intraoperatively (13.06mg±14.71 *versus* 25.09mg±22.50; p<0.001), at the PACU (4.50mg±8.05 *versus* 8.70mg±9.76; p=0.0012), and at 48 hours (83.07mg±53.78 *versus* 131.51mg±159.54; p=0.0093) ( Tables 1 to 3 [Table t2] [Table t3] ; Figure 2 ). Opioid requirements were significantly lower at all time points studied when patients were analyzed by subgroups that had either spinal or general anesthesia during the surgical procedure.

**Table 1:** Total postoperative opioid use

Total postoperative opioid use	QLB (n=79) Mean±SD (mg)*	No block (n=159) Mean±SD (mg)*	p value
Intraoperative	13.06±14.71	25.09± 22.50	<0.001
At the PACU	4.50±8.05	8.70± 9.76	0.0012
24 hours	53.82±37.41	77.59± 58.42	0.0011
48 hours	83.07±٥53.78	131.51± 159.54	0.0093

* in milligram oral morphine equivalent units.

QLB: quadratus lumborum block; SD: standard deviation; PACU: post anesthesia care.

**Table 2 t2:** Total postoperative opioid use in spinal anesthesia cases

Total postoperative opioid use in spinal anesthesia cases	QLB (n=79) Mean±SD (mg)*	No block (n=159) Mean±SD (mg)*	p value
Intraoperative	6.93±3.52	10.16±6.25	<0.001
At the PACU	1.74±2.64	2.32±3.85	<0.001
24 hours	42.39±28.24	57.08±35.68	0.0020
48 hours	66.52±54.42	144.94±110.86	0.0039

* In milligram oral morphine equivalent units.

QLB: quadratus lumborum block; SD: standard deviation; PACU: post anesthesia care.

**Table 3 t3:** Total postoperative opioid use in general anesthesia cases

Total postoperative opioid use in general anesthesia cases	QLB (n=79) Mean±SD (mg)*	No block (n=159) Mean±SD (mg)*	p value
Intraoperative	22.81±14.66	27.19±16.34	<0.001
At the PACU	8.97±9.62	9.73±10.22	<0.001
24 hours	71.68±43.35	80.90±48.96	0.0432
48 hours	109.49±60.63	129.35±88.27	0.0339

* In milligram oral morphine equivalent units.

QLB: quadratus lumborum block; SD: standard deviation; PACU: post anesthesia care.

**Figure 3:**
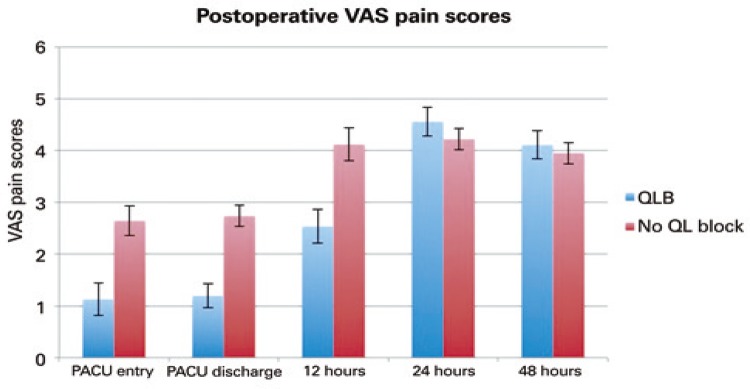
Postoperative Visual Analog Scale Pain scores, at different time points, for patients undergoing total hip arthroplasty (with standard error bars)

VAS: Visual Analog Scale; QLB: quadratus lumborum block; PACU: post anesthesia care.

**Figure 4:**
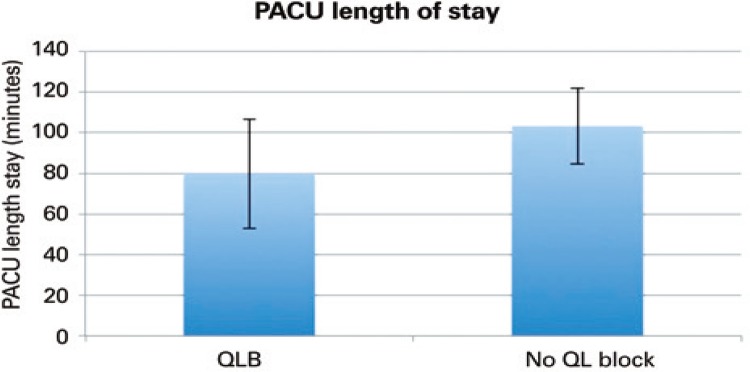
Post-anesthesia care unit length of stay (minutes) for patients undergoing total hip arthroplasty (with standard error bars)

PACU: post-anesthesia care; QLB: quadratus lumborum block.

Patient-reported VAS pain scores were lower at PACU admission (1.13 *versus* 2.65; p=0.0012), PACU discharge (1.20 *versus* 2.74; p<0.0001), and 12 hours after surgery (2.54 *versus* 4.12; p=0.0021) for patients who received a block ( Tables 4 to 6 [Table t5] [Table t6] ). There was no significant difference in pain scores at 24 hours (4.56 *versus* 4.22; p=0.359) or 48 hours (4.11 *versus* 3.95; p=0.704) postoperatively ( Figure 3 ). Pain VAS scores were significantly lower at PACU admission, PACU discharge and at 12 hours postoperatively, when patients were analyzed by subgroups that had either spinal or general anesthesia during the surgical procedure ( Table 2 ). PACU length of stay for patients who underwent THA under general anesthesia was significantly shorter in the QLB group when compared to the control group ( Table 7 , Figure 4 ).

**Table 4:** Total postoperative Visual Analog Scale pain score

Total postoperative VAS pain score	QLB (n=79) Mean±SD	No block (n=159) Mean±SD	p value
PACU admission	1.13±2.77	2.65±3.65	0.0012
PACU discharge	1.20±2.07	2.74±2.57	<0.001
12 hours	2.54±2.88	4.12±3.98	0.0021
24 hours	4.56±2.47	4.22±2.63	0.359
48 hours	4.11±2.40	3.95±2.63	0.704

VAS: Visual Analog Scale; QLB: quadratus lumborum block; SD: standard deviation; PACU: post anesthesia care.

**Table 5 t5:** Total postoperative Visual Analog Scale pain score for spinal anesthesia cases

Total postoperative VAS pain score in spinal anesthesia	QLB (n=79) Mean±SD	No block (n=159)Mean±SD	p value
PACU admission	0.085±0.054	0.078±0.062	0.485
PACU discharge	0.928±0. 38	1.000±0.26	<0.001
12 hours	1.55±2.68	3.095±2.53	0.0012
24 hours	4.524±2.85	4.105±3.29	0.8152
48 hours	4.357±2.42	3.0±3.88	0.3674

VAS: Visual Analog Scale; QLB: quadratus lumborum block; SD: standard deviation; PACU: post anesthesia care.

**Table 6 t6:** Total postoperative Visual Analog Scale pain score for general anesthesia cases

Total postoperative VAS pain score in general anesthesia	QLB (n=79) Mean±SD	No block (n=159) Mean±SD	p value
PACU admission	2.742±2.20	3.081±2.85	<0.001
PACU discharge	1.968±2.44	3.022±2.95	<0.001
12 hours	2.893±2.88	4.328±3.53	0.0082
24 hours	4.643±2.74	4.235±2.98	0.8152
48 hours	3.88±3.35	4.091±2.97	0.3674

VAS: Visual Analog Scale; QLB: quadratus lumborum block; SD: standard deviation; PACU: post anesthesia care.

**Table 7:** Post-anesthesia care unit length of stay for general anesthesia cases

PACU length of stay for general anesthesia cases	QLB (n=32) Mean±SD (minutes)	No block (n=137) Mean±SD (minutes)	p value
PACU length of stay (minutes)	79.77±239.68	103.38±236.77	0.0085

PACU: post-anesthesia care; QLB: quadratus lumborum block; SD: standard deviation.

## DISCUSSION

We studied the effects of preoperative posterior QLB for primary THA, chiefly regarding total opioid consumption, and we found that patients who received posterior QLB in both subsets (general anesthesia or spinal anesthesia), had significantly less opioid consumption when compared to the Control Group. Additionally, VAS pain scores in the QLB group in both subsets were significantly lower during PACU stay (admission and discharge) and for the first 12-hour period. Interestingly, VAS scores were not significantly different at 24 hours and 48 hours between the two groups. This study supports existing evidence that QLB result in analgesia after THA.^7 - 12^ Lower opioid consumption could have contributed to the shorter PACU length of stay observed in the posterior QLB group.

We performed posterior QLB for THA at our organization, injecting the local anesthetic in the fascial plane lying on the posterior border of the quadratus lumborum muscle, between the quadratus lumborum muscle, sacrospinalis and latissimus dorsi muscles. There is little evidence suggesting that one QLB approach is better than others with respect to duration, spread or clinical effects. The effect and duration of QLB have been shown to extend up to 48 hours after injection of 150mg of ropivacaine for laparoscopic surgery.^16^ In our institutional clinical practice, 20mL of 0.25% bupivacaine with 1:400 concentration epinephrine is commonly used for posterior QLB. The conservative doses of local anesthetic used in our clinical practice may explain the duration of the analgesic benefit limited to 12 hours, as assessed by VAS pain scores. There may be a potential to increase the total dose of local anesthetic, to enhance the analgesic duration.

Less opioid consumption can also potentially result in fewer opioid related adverse effects, early participation in physical therapy, faster recovery and discharge. There is also a potential benefit of less dependence and addiction to opioids due to opioid sparing effects of the posterior QLB for THA. Although we are unable to definitively assess this theory from our data, randomized trials in the future may be able to assess these short-term and long-term benefits of reduced opioid consumption.

The unequal sample sizes in the two groups were statistically adjusted to optimize the power of the study. Demographics in both groups were similar, and data collection was performed using random sampling to avoid selection bias. This retrospective study has its own limitations. We did not assess the dermatomal levels of the block after block placement to assess its functionality. As this was a novel block in our practice, conservative doses of local anesthetic were used for safety purposes. The effects of posterior QLB on ambulation and motor weakness were not assessed in the present study due to lack of data, and this is an area for future study.

To the best of our knowledge, this is the first retrospective study comparing posterior QLB to a control group in patients undergoing THA. This retrospective study has shown obvious benefits of posterior QLB in providing effective analgesia, and in reducing opioid consumption after primary THA. A randomized blinded prospective study is warranted to further investigate the analgesic effect and safety profile of QLB for THA.

## CONCLUSION

Posterior quadratus lumborum blocks for primary total hip arthroplasty is associated with decreased opioid requirements up to 48 hours. Preoperative posterior quadratus lumborum blocks for total hip arthroplasty decreases Visual Analog Scale pain scores up to 12 hours, and shortens post-anesthesia care unit length of stay. In sum, this study provides evidence that posterior quadratus lumborum block improves postoperative analgesia after total hip arthroplasty in an opioid-sparing manner.
