# Supplementary material for: Posterior quadratus lumborum block for primary total hip arthroplasty analgesia: a comparative study
Source: Einstein (Sao Paulo). 2019 Sep 4;17(4):eAO4905. doi: 10.31744/einstein_journal/2019AO4905 (PMC6750883; doi:10.31744/einstein_journal/2019AO4905)
Supplement: Translation

# Translation: Bloqueio anestésico do quadrado lombar posterior para analgesia na artroplastia total do quadril primária: estudo comparativo

**Authors:** Promil Kukreja, Lisa MacBeth, William Potter, Katherine Buddemeyer, Henry DeBell, Hesham Elsharkawy, Hari Kalagara, Andre Wajnsztejn, Eduardo Araujo Pires, Alexandre Leme Godoy-Santos, Ashish Shah

**Affiliations:** 1 Department of Anesthesiology and Perioperative Medicine, University of Alabama, Birmingham, AL, USA.; 2 Department of Orthopaedic Surgery, University of Alabama, Birmingham, AL, USA.; 3 Cleveland Clinic, Anesthesiology Institute, Cleveland, OH, USA.; 4 Hospital Israelita Albert Einstein, São Paulo, SP, Brazil.; 5 Department of Orthopedics and Trauma, Hospital das Clinicas, Faculdade de Medicina, Universidade de São Paulo, São Paulo, SP, Brazil.

**Keywords:** Analgesia, Anestésicos locais, Artroplastia de quadril, Dor pós-operatória, Analgésicos opioides

## Abstract

**Objetivo:**

Comparar a analgesia e o uso de opioides em pacientes submetidos à artroplastia total do quadril primária com bloqueio pré-operatório do quadrado lombar posterior e pacientes que não receberam o bloqueio do quadrado lombar.

**Métodos:**

Revisamos os prontuários de pacientes submetidos à artroplastia total do quadril unilateral entre 1º de janeiro de 2017 e 31 de março de 2018, e 238 pacientes foram incluídos no estudo. O desfecho primário foi o consumo de opioides no pós-operatório nas primeiras 24 horas. Os desfechos secundários foram consumo de opioide no intraoperatório, na sala de recuperação pós-anestésica e nas primeiras 48 horas, escores de Escala Visual Analógica de dor pós-operatória, e tempo de permanência na recuperação pós-anestésica. Os desfechos primário e secundários foram comparados entre os pacientes submetidos à artroplastia total do quadril primária com bloqueio pré-operatório do quadrado lombar posterior e aqueles que não receberam o bloqueio do quadrado lombar.

**Resultados:**

Para o grupo que recebeu o bloqueio, as doses totais de morfina por via oral em 24 horas foram menores (53,82mg±37,41) em comparação ao grupo sem bloqueio (77,59mg±58,42), com p=0,0011. A utilização de opioides foi consistentemente menor para o grupo que recebeu o bloqueio em cada tempo adicional de avaliação até 48 horas. Os escores da Escala Visual Analógica até 12 horas após a cirurgia para os pacientes que receberam o bloqueio do quadrado lombar posterior e o tempo de permanência na sala de recuperação pós-anestésica foram menores para o grupo que recebeu o bloqueio.

**Conclusão:**

O bloqueio anestésico do quadrado lombar posterior para artroplastia total do quadril primária está associado à diminuição do uso de opioides nas primeiras 48 horas, diminuição do escore de dor da Escala Visual Analógica em até 12 horas, e menor tempo de permanência na sala de recuperação pós-anestésica. Nível de evidência: III

## INTRODUÇÃO

A artroscopia total do quadril (ATQ) é um dos procedimento ortopédicos mais comuns nos Estados Unidos, com aproximadamente 300 mil cirurgias realizadas anualmente e cujos números devem aumentar com o envelhecimento da população.^1^ Na era dos protocolos *fast-track* para artroplastias totais, não parece haver um padrão-ouro para o manejo da dor no pós-operatório.^1^

O bloqueio do quadrado lombar (BQL) foi descrito pela primeira vez em 2007 como bloqueio posterior do plano transverso abdominal (PTA) para analgesia satisfatória pós-abdominoplastia.^2^ Existem muitas técnicas de BQL, com a deposição do anestésico local lateral, posterior ou anteriormente (transmuscular) ao músculo quadrado lombar.^3^

Desde a descrição inicial, o BQL tem sido usado para analgesia pós-operatória de cirurgias abdominais, incluindo cesarianas, herniorrafia inguinal e laparotomia.^4 - 6^ Além disso, existem relatos de casos de analgesia pós-operatória satisfatória após ATQ.^7 - 11^ Em pacientes submetidos à hemiartroplastia para fratura de colo do fêmur, o BQL lateral obteve escores de Escala Visual Analógica (EVA) de dor mais baixos, e consumo menor de opioide comparado ao bloqueio do nervo femoral.^12^ Os possíveis mecanismos de ação subjacentes do BQL levando à analgesia de quadril podem ser por conta da dispersão do anestésico local para as raízes nervosas e ramos do plexo lombar.^13 - 15^

## OBJETIVO

Avaliar os efeitos clínicos na analgesia de pacientes submetidos ao bloqueio posterior do quadrado lombar no pré-operatório de artroplastia total de quadril primária, considerando-se o consumo de opioide e o escore da escala visual analógica de dor.

## MÉTODOS

Este estudo retrospectivo foi aprovado pelo Comitê de Ética em Pesquisa (300000976) de acordo com a Declaração de Helsinki. Os registros de cobrança hospitalares de nossa instituição, a *University of Alabama,* em Birmingham, foram utilizados para localizar todas as artroplastias totais do quadril, realizadas entre 1^o^de janeiro de 2017 e 31 de março de 2018, por meio dos códigos do Sistema de Codificação de Procedimento da Classificação Estatística Internacional de Doenças e Problemas Relacionados com a Saúde (SCP CID-10). A busca identificou 559 casos, e a amostragem aleatória foi usada para selecionar os pacientes. Os casos submetidos à revisão de ATQ, à abordagem anterior para ATQ e aos BQL no pós-operatório foram excluídos, permanecendo somente 355 pacientes submetidos à ATQ primária, com ou sem BQL posterior antes da cirurgia. Além disso, 117 pacientes com dados incompletos foram excluídos do estudo, permanecendo 238 prontuários analisados.

O presente estudo comparou pacientes submetidos ao BQL posterior antes de ATQ primária com pacientes submetidos à ATQ primária sem bloqueio. Dados referentes ao tipo de anestesia usado, presença ou ausência de BQL posterior, momento de admissão na unidade de recuperação pós-anestésica (RPA) e momento de alta da RPA também foram coletados. Os escores de dor da EVA de zero a 10, no pós-operatório durante a internação, e o consumo de opioide, durante a internação, foram coletados até 48 horas após cirurgia nos seguintes momentos: admissão na RPA, alta da RPA, 24, e 48 horas após o início da cirurgia. O consumo total de opioide foi convertido em unidades equivalentes (mg) de morfina oral. Os escores EVA de dor mais próximos aos momentos de interesse foram coletados. O desfecho primário do estudo foi o consumo de opioide nas 24 horas do pós-operatório. Os desfechos secundários incluídos foram consumo de opioide no intraoperatório, na RPA e nas 48 horas do pós-operatório; escores da EVA de dor na admissão e alta da RPA, 12, e 48 horas do pós-operatório; e tempo de permanência na RPA.

Os BQL posteriores foram realizados na área pré-operatória de bloqueio em decúbito lateral, com monitorização padrão de oximetria de pulso, monitor de pressão arterial não invasiva e eletrocardiograma. Foi utilizada sonda curvilínea de baixa frequência ( Figura 1 ), para o BQL posterior, usando abordagem lateromedial, em plano, com 20mL de bupivacaína 0,25% com adrenalina na concentração de 1:400. Os pacientes foram, então, submetidos à ATQ, sob anestesia geral ou raquianestesia.

**Figura 1:** Abordagem guiada por ultrassom para bloqueio do quadrado lombar posterior. A seta branca indica a borda posterior do músculo quadrado lombar

EO: *external oblique* ; IO: *internal oblique* ; TA: *transversus abdominis* ; QL: *quadratus lumborum* .

Todas as variáveis demográficas e clínicas com medidas contínuas foram expressas como médias e desvios padrão; variáveis categóricas foram expressas em proporções. Para dados de distribuição não normal, foi usado o programa NPAR1WAY SAS para testes não paramétricos para realizar análise de variância padrão. Os tamanhos desiguais de amostra foram ajustados para maximizar o poder estatístico. A distribuição das variáveis contínuas foi estudada usando o teste Kolmogorov-Smirnov. Para dados com distribuição normal, a Análise de Variância (ANOVA) *one-way* e o teste *t* de Student foram usados para comparar os grupos de dados. Foram usados o teste Kruskal-Wallis e o teste de Wilcoxon para comparar dados que geralmente não apresentam distribuição normal. O teste χ^2^ e o teste exato de Fisher foram usados para analisar as variáveis categóricas. Foram considerados estatisticamente significantes valores de p≤0,05 (bicaudal) para todas as comparações. O teste *t* de Student e a ANOVA foram usados para comparar o consumo de opioide, os escores EVA e tempo de permanência na RPA.

## RESULTADOS

O estudo avaliou 238 pacientes, sendo 79 submetidos ao BQL e 159 não submetidos ao bloqueio. Para os pacientes submetidos ao bloqueio posterior do quadrado lombar, a dose total equivalente de morfina oral (mg) consumida em 24 horas foi 53,82mg±37,41, em comparação ao Grupo Controle, em que foi de 77,59mg±58,42 (média± desvio padrão − DP), com p=0,0011. Os valores de opioide necessários foram significativamente menores para os pacientes submetidos ao BQL, de forma consistente em cada momento estudado: intraoperatório (13,06mg±14,71 *versus* 25,09mg±22,50; p<0,001), na RPA (4,50mg±8,05 *versus* 8,70mg±9,76; p=0,0012), e nas 48 horas (83,07mg±53,78 *versus* 131,51mg±159,54; p=0,0093) ( Tabelas 1 a 3 [Table t2002] [Table t3002] ; Figura 2 ). O consumo de opioides foi significativamente menor em todos os momentos estudados ao se analisarem os pacientes por subgrupo de raquianestesia, ou anestesia geral durante o procedimento cirúrgico.

**Tabela 1:** Total de opioide no pós-operatório

Total de opioide no pós-operatório	BQL (n=79) Média±DP (mg)*	Sem bloqueio (n=159) Média±DP (mg)*	Valor de p
Intraoperatório	13,06±14,71	25,09±22,50	<0,001
Na RPA	4,50±8,05	8,70±9,76	0,0012
24 horas	53,82±37,41	77,59±58,42	0,0011
48 horas	83,07±53,78	131,51±159,54	0,0093

* Unidades equivalentes de morfina oral em miligramas.

BQL: bloqueio do quadrado lombar; DP: desvio padrão; RPA: recuperação pós-anestésica.

**Tabela 2 t2002:** Total de opioide no pós-operatório em casos de raquianestesia

Total opioide pós-operatório em casos de raquianestesia	BQL (n=79) Média±DP (mg)*	Sem bloqueio (n=159) Média±DP (mg)*	Valor de p
Intraoperatório	6,93±3,52	10,16±6,25	<0,001
Na RPA	1,74±2,64	2,32±3,85	<0,001
24 Horas	42,39±28,24	57,08±35,68	0,0020
48 Horas	66,52±54,42	144,94±110,86	0,0039

* Unidades equivalentes de morfina oral em miligramas.

BQL: bloqueio do quadrado lombar; DP: desvio padrão; RPA: recuperação pós-anestésica.

**Tabela 3 t3002:** Total de opioide no pós-operatório nos casos de anestesia geral

Total de opioide no pós-operatório em casos de anestesia geral	BQL (n=79) Média±DP (mg)*	Sem Bloqueio (n=159) Média±DP (mg)*	Valor de p
Intraoperatório	22,81±14,66	27,19±16,34	<0,001
Na RPA	8,97±9,62	9,73±10,22	<0,001
24 Horas	71,68±43,35	80,90±48,96	0,0432
48 Horas	109,49±60,63	129,35±88,27	0,0339

* unidades equivalentes de morfina oral em miligramas.

BQL: bloqueio do quadrado lombar; DP: desvio padrão; RPA: recuperação pós-anestésica.

**Figura 2:** Consumo total de opioide em unidades equivalentes de morfina oral (mg) em diferentes momentos para pacientes submetidos à artroplastia total do quadril (com barras de erro padrão)

RPA: recuperação pós-anestésica; BQL: bloqueio do quadrado lombar.

No grupo submetido a bloqueio, os escores de dor segundo a EVA referidos pelos pacientes foram menores na admissão à RPA (1,13 *versus* 2,65; p=0,0012), na alta da RPA (1,20 *versus* 2,74; p<0,0001) e 12 horas após a cirurgia (2,54 *versus* 4,12; p=0,0021) ( Tabelas 4 a 6 [Table t5002] [Table t6002] ). Não houve diferenças significativas nas escalas de dor nas 24 horas (4,56 *versus* 4,22; p=0,359) ou 48 horas (4,11 *versus* 3,95; p=0,704) pós-operatórias ( Figura 3 ). A EVA foi significativamente menor na admissão na RPA, na alta da RPA e nas 12 horas após a cirurgia, quando os pacientes foram analisados por subgrupo em termos de raquianestesia ou anestesia geral durante o procedimento cirúrgico ( Tabela 2 ). O tempo de permanência na RPA para pacientes submetidos à ATQ sob anestesia geral foi significativamente menor para o grupo submetido ao BQL em comparação ao controle ( Tabela 7 , Figura 4 ).

**Tabela 4:** Pontuação total na Escala Visual Analógica de dor no pós-operatório

Pontuação total na EVA de dor no pós-operatório	BQL (n=79)Média±DP	Sem bloqueio (n=159) Média±DP	Valor de p
Admissão na RPA	1,13±2,77	2,65±3,65	0,0012
Alta da RPA	1,20±2,07	2,74±2,57	<0,001
12 horas	2,54±2,88	4,12±3,98	0,0021
24 horas	4,56±2,47	4,22±2,63	0,359
48 horas	4,11±2,40	3,95±2,63	0,704

EVA: Escala Visual Analógica; BQL: bloqueio do quadrado lombar; DP: desvio padrão; RPA: recuperação pós-anestésica.

**Tabela 5 t5002:** Pontuação total na Escala Visual Analógica de dor no pós-operatório, em casos de raquianestesia

Pontuação total na EVA de dor no pós-operatório, em casos de raquianestesia	BQL (n=79) Média±DP	Sem bloqueio (n=159) Média±DP	Valor de p
Admissão na RPA	0,085±0,054	0,078±0,062	0,485
Alta da RPA	0,928±0,38	1,000±0,26	<0,001
12 horas	1,55±2,68	3,095±2,53	0,0012
24 horas	4,524±2,85	4,105±3,29	0,8152
48 horas	4,357±2,42	3,0±3,88	0,3674

EVA: Escala Visual Analógica; BQL: bloqueio do quadrado lombar; DP: desvio padrão; RPA: recuperação pós-anestésica.

**Tabela 6 t6002:** Pontuação total na Escala Visual Analógica de dor no pós-operatório, em casos de anestesia geral

Pontuação total na EVA de dor no pós-operatório, em casos de anestesia geral	BQL (n=79) Média ± DP	Sem bloqueio (n=159) Média ± DP	Valor de p
Admissão na RPA	2,742±2,20	3,081±2,85	<0,001
Alta da RPA	1,968±2,44	3,022±2,95	<0,001
12 horas	2,893±2,88	4,328±3,53	0,0082
24 horas	4,643±2,74	4,235±2,98	0,8152
48 horas	3,88±3,35	4,091±2,97	0,3674

EVA: Escala Visual Analógica; BQL: bloqueio do quadrado lombar; DP: desvio padrão; RPA: recuperação pós-anestésica.

**Figura 3:** Pontuação na Escala Visual Analógica de dor no pós-operatório, em diferentes momentos, para pacientes submetidos à artroplastia total de quadril (com barras de erro padrão)

EVA: Escala Visual Analógica; BQL: bloqueio do quadrado lombar; RPA: recuperação pós-anestésica.

**Tabela 7:** Tempo de permanência na unidade de recuperação pós-anestésica para casos de anestesia geral

Tempo de permanência na RPA para casos de anestesia geral	BQL (n=32) Média±DP (minutos)	Sem bloqueio (n=137) Média±DP (minutos)	Valor de p
Tempo de internação na RPA (minutos)	79,77±239,68	103,38±236,77	0,0085

RPA: recuperação pós-anestésica; BQL: bloqueio do quadrado lombar.

**Figura 4:** Tempo de permanência na unidade de recuperação pós-anestésica (minutos) para pacientes submetidos à artroplastia total de quadril (com barras de erro padrão)

RPA: recuperação pós-anestésica; BQL: bloqueio do quadrado lombar.

## DISCUSSÃO

Estudamos os efeitos do BQL posterior pré-operatório na ATQ primária, especialmente em relação ao consumo total de opioide, e observamos que os pacientes que receberam tal bloqueio em ambos os subconjuntos (anestesia geral ou raquianestesia) tiveram consumo de opioide significativamente menor em comparação aos controle. Além disso, a pontuação na EVA de dor no grupo BQL em ambos os subconjuntos foram significativamente menores durante a permanência na RPA (admissão e alta) e no período inicial de 12 horas. É interessante a observação de que os escores da EVA não foram significativamente diferentes nas 24 horas e 48 horas entre os dois grupos. O estudo corrobora a evidência existente de que o BQL resulta em analgesia após ATQ.^7 - 12^ O menor consumo de opioide pode ter contribuído para o menor tempo de permanência na RPA observado no grupo com BQL posterior.

Na nossa instituição, realizamos BQL posterior para ATQ injetando o anestésico local no plano fascial da borda posterior do músculo quadrado lombar, entre o músculo quadrado lombar, e os músculos sacroespinal e grande dorsal. Existe pouca evidência que sugira a superioridade de uma técnica de BQL sobre as demais em relação a duração, dispersão ou efeitos clínicos. Já se demonstrou que o efeito e duração do BQL se estendem até 48 horas após injetar 150mg de ropivacaína para cirurgia laparoscópica.^16^ Na prática clínica da nossa instituição, 20mL de bupivacaína a 0,25% com adrenalina na concentração de 1:400 são usualmente usados para BQL posterior. As doses conservadoras de anestésico local usadas na nossa prática clínica podem explicar a duração do benefício anestésico limitado a 12 horas, segundo a avaliação feita usando-se a EVA de dor. Existe a possibilidade potencial de aumentar a dose total de anestésico local e prolongar a duração da analgesia.

O menor consumo de opioide pode também potencialmente resultar em menos efeitos adversos relacionados a opioide, introdução precoce de fisioterapia, e recuperação e alta mais rápidas. Também existe o benefício potencial de menor dependência ou vício aos opioides, pelos efeitos poupadores deles, ao empregarmos o BQL posterior para ATQ. Embora não tenhamos conseguido avaliar essa teoria de forma definitiva a partir de nossos dados, estudos randomizados no futuro podem conseguir avaliar esses benefícios a curto e longo prazo do consumo reduzido de opioides.

Os tamanhos desiguais das amostras dos dois grupos foram ajustados estatisticamente para otimizar o poder do estudo. As características demográficas em ambos os grupos foram semelhantes, e a coleta de dados foi feita usando técnica de amostragem aleatória para evitar viés de seleção. O presente estudo retrospectivo possui limitações. Não avaliamos os níveis de dermátomo do bloqueio após o estabelecimento do bloqueio para avaliar a funcionalidade do mesmo. Como se tratava de bloqueio novo na nossa prática, empregamos dose conservadora de anestésico local por razões de segurança. Os efeitos do BLQ posterior na deambulação e na fraqueza motora não foram avaliados neste estudo por falta de dados, mas podem ser objeto de estudo futuro.

Até onde sabemos, o presente estudo foi o primeiro retrospectivo comparando BQL posterior a Grupo Controle em pacientes submetidos à ATQ. Este estudo retrospectivo mostrou evidentes benefícios do BQL posterior para estabelecer analgesia efetiva e reduzir o consumo pós-operatório de opioide em pacientes submetidos à ATQ primária. Um estudo randomizado cego prospectivo é necessário para aprofundar a investigação do efeito analgésico e o perfil de segurança do BQL na ATQ.

## CONCLUSÃO

O bloqueio do quadrado lombar posterior para artroplastia total de quadril é associado a menor necessidade de opioide até 48 horas. O bloqueio posterior pré-operatório do quadrado lombar para artroplastia total de quadril diminuiu os escores da Escala Visual Analógica de dor em até 12 horas e reduziu o tempo de permanência na sala de recuperação anestésica. Em suma, o presente estudo fornece evidência de que o bloqueio posterior do quadrado lombar melhora a analgesia pós-operatória depois de artroplastia total de quadril, de forma a poupar o consumo de opioide.
